# Dependence of resting-state-based cerebrovascular reactivity (CVR) mapping on spatial resolution

**DOI:** 10.3389/fnimg.2023.1205459

**Published:** 2023-06-26

**Authors:** Peiying Liu, Beini Hu, Lincoln Kartchner, Parimal Joshi, Cuimei Xu, Dengrong Jiang

**Affiliations:** ^1^Department of Diagnostic Radiology and Nuclear Medicine, University of Maryland School of Medicine, Baltimore, MD, United States; ^2^Department of Radiology, Johns Hopkins University School of Medicine, Baltimore, MD, United States

**Keywords:** cerebrovascular reactivity (CVR), resting-state fMRI, resolution-dependence, duration-dependence, reproducibility

## Abstract

Cerebrovascular reactivity (CVR) is typically assessed with a carbon dioxide (CO_2_) stimulus combined with BOLD fMRI. Recently, resting-state (RS) BOLD fMRI has been shown capable of generating CVR maps, providing a potential for broader CVR applications in neuroimaging studies. However, prior RS-CVR studies have primarily been performed at a spatial resolution of 3–4 mm voxel sizes. It remains unknown whether RS-CVR can also be obtained at high-resolution without major degradation in image quality. In this study, we investigated RS-CVR mapping based on resting-state BOLD MRI across a range of spatial resolutions in a group of healthy subjects, in an effort to examine the feasibility of RS-CVR measurement at high resolution. Comparing the results of RS-CVR with the maps obtained by the conventional CO2-inhalation method, our results suggested that good CVR map quality can be obtained at a voxel size as small as 2 mm isotropic. Our results also showed that, RS-CVR maps revealed resolution-dependent sensitivity. However, even at a high resolution of 2 mm isotropic voxel size, the voxel-wise sensitivity is still greater than that of typical task-evoked fMRI. Scan duration affected the sensitivity of RS-CVR mapping, but had no significant effect on its accuracy. These findings suggest that RS-CVR mapping can be applied at a similar resolution as state-of-the-art fMRI studies, which will broaden the use of CVR mapping in basic science and clinical applications including retrospective analysis of previously collected fMRI data.

## Introduction

Cerebrovascular reactivity (CVR), an index of the dilatory function of cerebral blood vessels, is a sensitive marker of cerebrovascular function and is increasingly used in studies of brain diseases such as stroke (Krainik et al., 2005; Geranmayeh et al., 2015), small vessel disease (Marstrand et al., 2002; Greenberg, 2006), dementia (Silvestrini et al., 2006; Cantin et al., 2011; Yezhuvath et al., 2012; Sur et al., 2020), Moyamoya disease (Mikulis et al., 2005; Donahue et al., 2013), traumatic brain injury (Chan et al., 2015; Kenney et al., 2016), and brain tumor (Pillai and Zaca, 2011; Zaca et al., 2014; Fierstra et al., 2016). MRI measurement of CVR is typically performed by applying a vasodilatory stimulus, often carbon dioxide (CO_2_) inhalation, while acquiring blood oxygenation level-dependent (BOLD) fMRI (Liu et al., 2019). However, the requirement of CO2 delivery inside the MRI scanner has been a long-standing obstacle preventing CVR from being used as routinely as some of the other MRI techniques such as perfusion MRI.

Recently, an emerging CVR technique, based on resting-state (RS) BOLD fMRI, has been shown capable of generating CVR maps without the need for a gas challenge (Golestrani et al., 2016; Liu et al., 2017, 2021). This new method exploits the spontaneous fluctuations in arterial CO2 level at rest, and provides an opportunity for broader applications of CVR MRI in neuroimaging studies (Liu et al., 2021), including retrospective analysis of RS-fMRI data in large-scale multi-site studies such as the Human Connectome Project (Smith et al., 2013) or UK Biobank (Miller et al., 2016). However, compared with CO_2_-CVR, resting-state CVR (RS-CVR) is known to suffer from a lower signal-to-noise ratio (SNR) due to a smaller amplitude of arterial CO2 fluctuation at rest when compared to that associated with CO2 inhalation. As a result, prior RS-CVR studies have primarily been performed at a spatial resolution of 3–4 mm voxel sizes (Golestrani et al., 2016; Liu et al., 2017, 2021; Taneja et al., 2019). As the fMRI field marches toward higher spatial resolutions, it remains unknown whether RS-CVR can also be obtained at high resolution without a major degradation in image quality.

Therefore, the goal of the present study is to investigate the effect of spatial resolution of the RS-BOLD sequence on RS-CVR mapping. RS-BOLD scans were performed at three resolutions at decreasing voxel size. The resulting CVR maps were compared against maps obtained with the conventional CO2-inhalation method.

## Methods

### Participants

Eight participants (3 females, 5 males, age 26.5 ± 5 years) in good general physical health were recruited and screened for potentially confounding vascular, neurological or psychiatric disorders, or MRI and protocol contraindications. The institutional review board of the Johns Hopkins University School of Medicine approved this Health Insurance Portability and Accountability Act–compliant study, and all data were obtained with participants' written informed consent.

### MRI acquisition

Images were acquired on a 3 Tesla Siemens Prisma scanner (Siemens Healthineers, Erlangen, Germany) using a 32-channel head coil for reception. After localizer acquisition, three RS-BOLD MRI runs were acquired in each subject, with a voxel size of 2 × 2 × 2 mm^3^, 2.4 × 2.4 × 2.4 mm^3^, and 3 × 3 × 3 mm^3^, respectively. The order of the three runs were randomized and balanced across subjects. Other BOLD imaging parameters were: TR/TE/FA = 720/37 ms/52°, multiband factor = 8, FOV = 208 × 208 × 144 mm^3^ (for 2 mm resolution), 210 × 210 × 155 mm^3^ (for 2.4 mm resolution), and 210 × 210 × 168 mm^3^ (for 3 mm resolution), number of measurement = 833, scan duration = 10 min. The FOVs were slightly different because the matrix size could only take a few discrete values. As a reference measure, CO2-inhalation CVR was also performed in which the participant inhaled room air for 15 s, followed by two repetitions of 50 s of CO2 gas mixture (5% CO2, 21% O2, and 74% N2) interleaved with 70 s of room air, and ended with another 45 s of room air inhalation. The CO2-delivery apparatus and procedure was described previously (Lu et al., 2014). Briefly, the participant was fitted with a nose-clip and a mouthpiece, so that they breathed with their mouth only. The CO2 gas mixture was delivered to the participant through a non-rebreathing valve from a Douglas bag. A researcher was inside the scanner room for the entire duration of the CO2-CVR scan to switch the gas valve and monitor the participant. The duration of the CO2-MRI scan was 5 min. The BOLD sequence in the CO2-CVR scan was based on the 2 mm protocol described above.

Additionally, for anatomic reference, a high-resolution T1-MPRAGE sequence was also acquired. The imaging parameters were: TR/TE/TI = 2,100/3.8/1,100 ms, FOV = 256 × 208 × 160 mm^3^, voxel size = 1 × 1 × 1 mm^3^, scan duration = 4 min.

### Data processing

The images were processed with Matlab software (Mathworks, Natick, MA) and SPM12 (https://www.fil.ion.ucl.ac.uk/spm/software/spm12/). The resting-state and CO_2_-inhalation BOLD data were analyzed following previously published methods (Lu et al., 2014; Liu et al., 2021). Specifically, for resting-state data, the pre-processing includes motion correction and detrending. The BOLD images were then temporally filtered to [0, 0.1164 Hz] based on reports described in Liu et al. (2021). A whole-brain mask was obtained using SPM_segmentation by segmenting the mean BOLD images and then combining gray and white matter masks. By applying the mask to the BOLD image series, a whole-brain-averaged time course was calculated. A voxel-by-voxel linear regression was then employed in which the whole-brain BOLD time course was the independent variable and the voxel-wise BOLD time course was the dependent variable. Motion vectors and a linear drift term were used as covariates. The coefficient of the linear regression model was used as a CVR index, which was then normalized to the whole-brain average of the index to yield a relative CVR map.

For the CO_2_-inhalation data, motion correction was first performed on the BOLD image series. In order to facilitate its comparison with all resting-state BOLD data, some of which were collected at varying spatial resolutions, the CO_2_-inhalation BOLD images (with an acquisition resolution of 2 × 2 × 2 mm^3^) were smoothed by 0, 1.33, and 2.24 mm Gaussian kernels to match the acquisition resolution of the 2, 2.4, and 3 mm resting-state scans, respectively. The smoothing step changed the smoothness of the CO2-inhalation BOLD images but maintained the original voxel size of 2 × 2 × 2 mm^3^. CO_2_-CVR maps were then computed for the three resolutions separately. The computation of CO_2_-CVR map followed a well-established method of linear regression between end-tidal CO_2_ (EtCO_2_) and BOLD time courses (Liu et al., 2019). Briefly, the EtCO_2_ time course was shifted to account for the lung-to-brain delay, and then the shifted EtCO_2_ was used as the independent variable in a voxel-wise regression analysis, generating an absolute CVR map in %ΔBOLD/mmHg CO_2_ which was then normalized to the whole-brain average CVR to yield a relative CVR map. All CVR maps were further co-registered to the image template of the Montreal Neurological Institute (MNI) via T1-MPRAGE images for the calculation of group-averaged CVR maps.

For the purpose of easy spatial comparison, the BOLD images of all resting-state scans were co-registered to the mean BOLD image of the CO_2_-CVR scan after motion correction, and re-sampled to a voxel size of 2 × 2 × 2 mm^3^. Therefore, all the resulting CVR maps from the same subjects were co-registered and voxel-wise comparisons can be performed directly across the scans.

### Statistical analysis

Sensitivity of the RS-CVR data at each spatial resolution was determined by the Z-score from the voxel-wise linear regression model fitting in RS-CVR map calculation. Histogram and mean Z-score of each resting-state data were calculated, and compared across different resolutions. Comparisons between resulting RS-CVR, CO2-CVR and Z-score maps were performed. Following the Shapiro-Wilks normality tests, comparisons were performed using one-way ANOVA with Tukey *post-hoc* tests for data with normal distribution.

Accuracy of the RS-CVR maps at each spatial resolution was determined by the spatial correspondence between the RS-CVR maps and the reference map of CO2-CVR. Specifically, Pearson's correlation coefficient (r) was computed between each RS-CVR map and the CO2-CVR map at the corresponding resolution.

Reproducibility was tested by separating out the first half of the resting-state BOLD scans from the second half, yielding two separate 5-min datasets from each scan. The image processing steps described above were performed separately on each of the 5-min scans and the resulting CVR maps were compared against each other to determine their spatial correlation (r). Spatial intraclass correlation (ICC) was also calculated to evaluate reproducibility of RS-CVR maps at each imaging resolution.

The effect of scan duration was also tested using the first 4, 5, 6, 7, 8, and 9 min of the resting-state BOLD scans for RS-CVR analysis, respectively, yielding another six CVR maps of different scan durations for each imaging resolution of each subject. In this analysis, an 8 mm FWHM smoothing kernel was applied on the BOLD images as recommended previously (Liu et al., 2022). Pearson's correlation coefficient (r) was calculated between each resulting RS-CVR maps and the CO2-CVR maps at the corresponding resolution. The r values and mean Z scores of the RS-CVR maps were compared across the imaging resolutions and scan durations using two-way repeated measures ANOVA. If significant effects or imaging resolution or scan duration were found, *post-hoc* pair-wise comparisons were performed using Tukey test with 95% family-wise confidence level.

All statistical analysis was performed using R 4.1.1 and Python 3.8.

## Results

**Figure 1:**
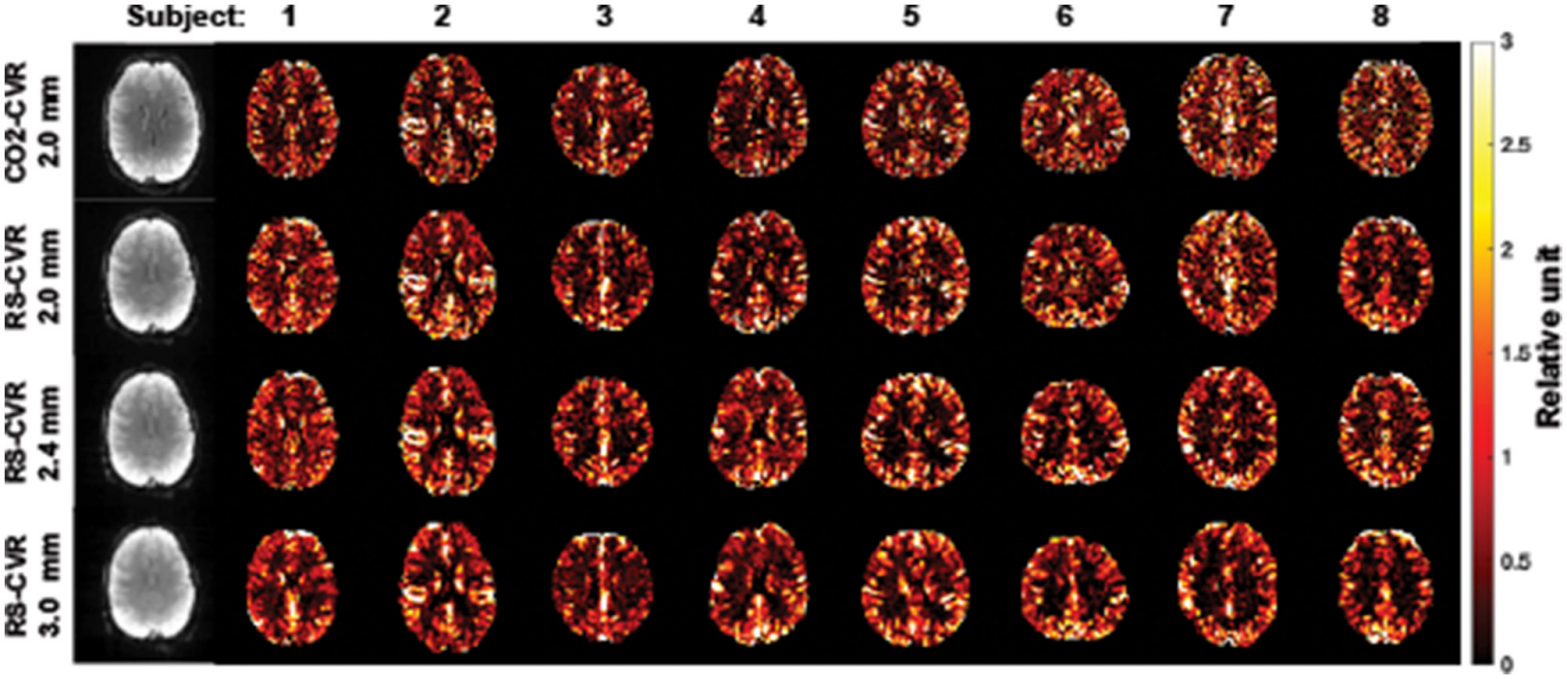
CVR maps for all subjects and imaging resolutions. The first column shows the raw BOLD images for subject 1.

**Figure 2:**
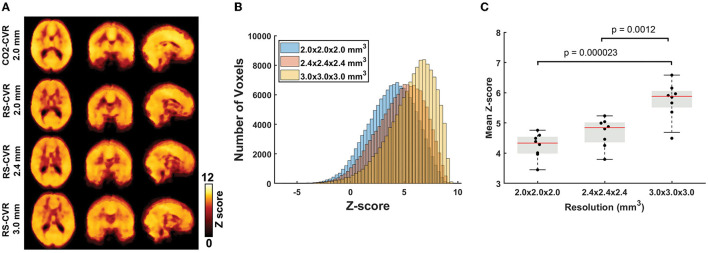
Results of the sensitivity analyses. **(A)** Group-averaged Z-maps of CO2-CVR and RS-CVR. **(B)** Histograms of group-average Z-scores of RS-CVR maps of the three imaging resolutions. **(C)** Comparisons of mean Z-scores of RS-CVR maps across different imaging resolutions.

Figure 1 shows the relative CVR maps obtained from each scan of each subject. Visual inspection suggested that the map quality is satisfactory in all scans, with clear gray/white matter contrast. Lower spatial resolution (i.e., larger voxel size) yielded images that are somewhat blurry but appear more stable. Figure 2A shows averaged Z map of each spatial resolution. Figure 2B shows the histograms of voxel-wise Z-scores with different spatial resolutions. Each histogram was based on all voxels of all subjects. Although the voxel-wise maps were not visually different significantly, the histograms suggested that the Z scores, which indicate the reliability of the linear regressions, increased with a larger voxel size. Boxplots of the mean Z-scores of the whole-brain are shown in Figure 2C. Since all data passed the normality tests (*p* > 0.05), parametric tests were used for statistical analysis. A significant main effect of spatial resolution was observed (*p* < 0.0001). *Post-hoc* analysis revealed that a resolution of 3 × 3 × 3 mm^3^ yielded a higher Z-score than scans with a resolution of 2 × 2 × 2 mm^3^ (*p* < 0.0001) or 2.4 × 2.4 × 2.4 mm^3^ (*p* = 0.0012). It should be noted that even at a resolution of 2 × 2 × 2 mm^3^, the mean Z-score across the brain is still above 4, suggesting that the coefficient estimated with linear regression was highly robust.

**Figure 3:**
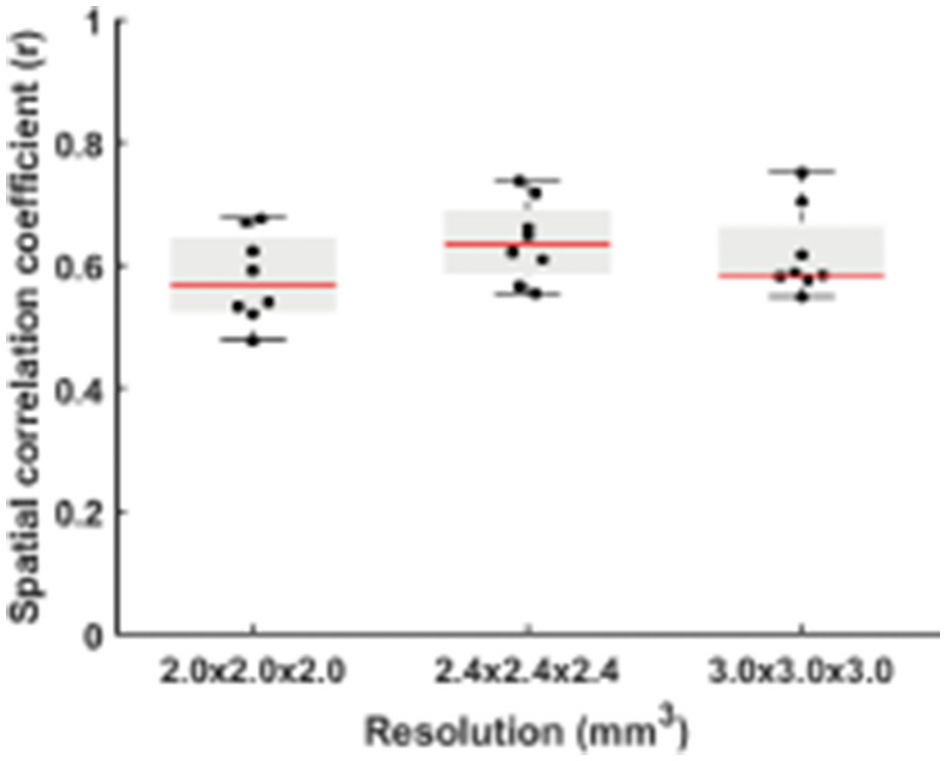
Comparisons of spatial correlation coefficients between the RS-CVR maps and CO2-CVR maps.

Figure 3 displays the spatial correlation coefficient (r) between RS-CVR and CO2-CVR as a function of three spatial resolutions. Although the correlation with the CO_2_-CVR maps was slightly lower for the resolution of 2 × 2 × 2 mm^3^ and slightly higher for the resolution of 2.4 × 2.4 × 2.4 mm^3^, ANOVA test showed no significant difference among different resolutions (*p* = 0.24). While these results were obtained with no spatial smoothing on the raw images, we also conducted additional analysis where the BOLD image series were pre-smoothed with 2, 4, 6, and 8 mm kernels, respectively. As shown in Supplementary Figure 1, two-way repeated measures ANOVA revealed that the overall spatial correlation between RS-CVR and CO2-CVR increased with larger smoothing kernels (*p* < 0.0001) but there was still not a significant difference across data of different resolutions. The degree of motion was also found to be not significantly different among the RS-CVR and CO2-CVR scans (*p* = 0.19).

**Figure 4:**
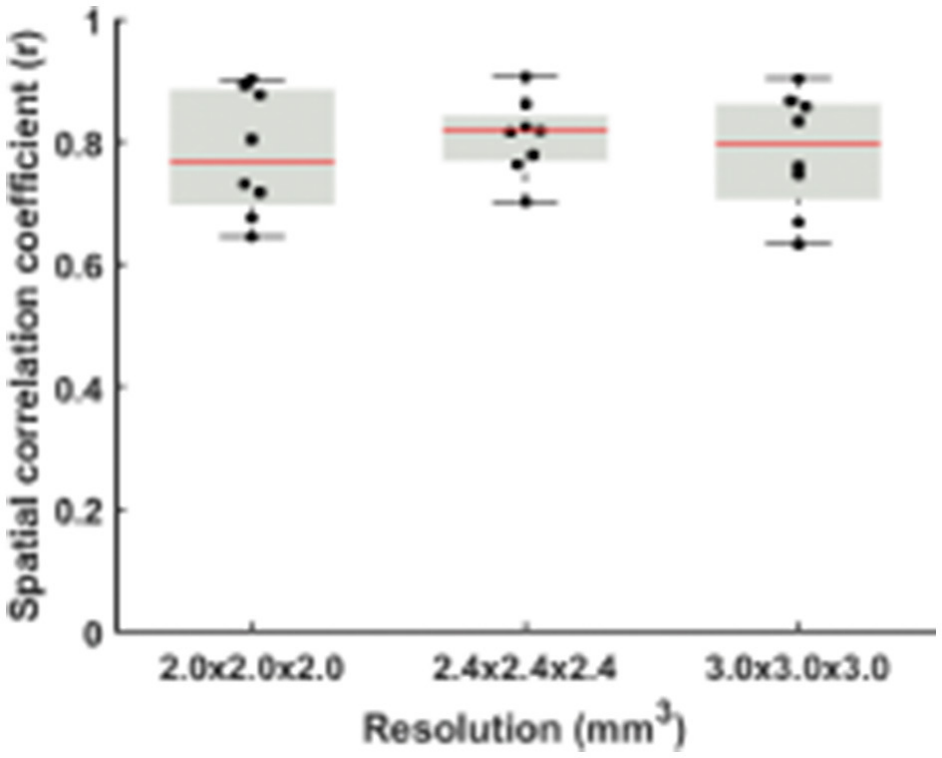
Comparisons of spatial correlation coefficients between RS-CVR maps obtained from the first and second halves of resting-state data.

When dividing the data from each run into two halves, the RS-CVR maps at all resolutions showed an excellent intra-session reproducibility (Figure 4). There was not a significant difference in reproducibility values across resolutions. The spatial ICC was 0.78 ± 0.10, 0.80 ± 0.06, and 0.78 ± 0.10 for 2 × 2 × 2 mm^3^, 2.4 × 2.4 × 2.4 mm^3^ and 3 × 3 × 3 mm^3^, respectively, also showing no significant difference across resolutions.

**Figure 5:**
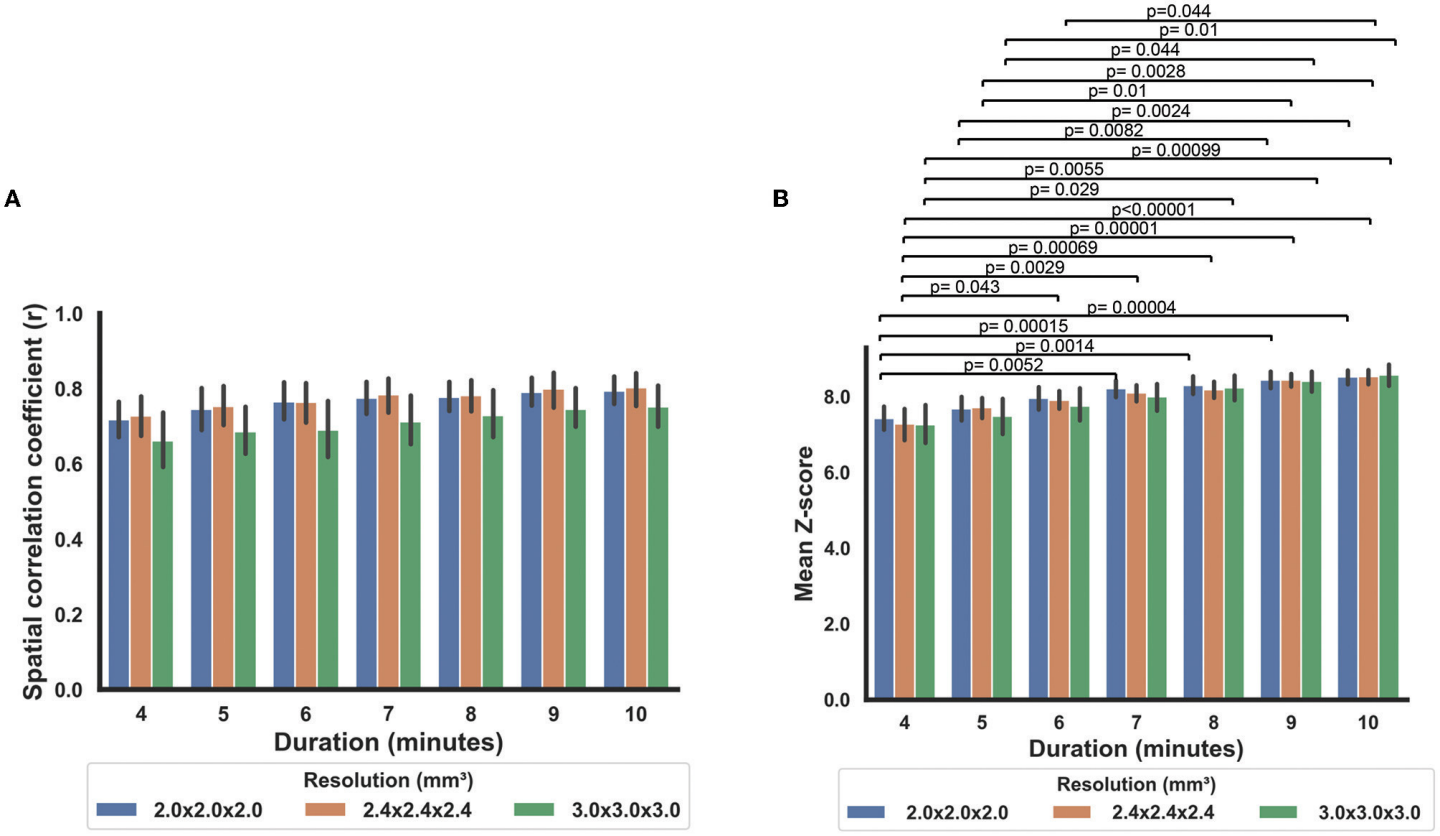
Comparisons of the RS-CVR maps at different scan durations. **(A)** Spatial correlation coefficients between the RS-CVR maps and CO2-CVR maps at different scan durations. **(B)** Mean Z-scores of the RS-CVR maps at different scan durations.

When comparing the data from different scan durations, the RS-CVR maps at all resolutions showed good spatial correlation with the CO2-CVR maps. Two-way repeated measures ANOVA allowed the evaluation of the effects of scan duration, imaging resolution and their interaction simultaneously. As shown in Figure 5, scan duration showed a significant effect on the sensitivity of RS-CVR maps with longer scans yielded better RS-CVR sensitivity (*p* < 0.0001), but not on the accuracy of the RS-CVR maps which was indicated as the r values with CO2-CVR maps. Imaging resolution did not show significant effects on both sensitivity (*p* = 0.68) and accuracy (*p* = 0.27), nor was there any interaction effects between imaging resolution and scan duration (*p* = 0.90 for sensitivity; *p* = 0.99 for accuracy). *Post-hoc* analyses showed that RS-CVR maps from the 4 and 5 min scans had significantly lower sensitivity than those from the 9 and 10 min scans (*p* < 0.05).

## Discussion

In this study, we investigated RS-CVR mapping based on resting-state BOLD MRI across a range of spatial resolutions, in an effort to examine the feasibility of RS-CVR measurement at high resolution. Comparing the results of RS-CVR with the maps obtained by the conventional CO2-inhalation method, our results suggested that good CVR map quality can be obtained at a voxel size as small as 2 mm isotropic, a resolution used in many recent fMRI reports and employed in several large-scale studies such as Human Connectome Project (Smith et al., 2013) and UK Biobank Study (Miller et al., 2016).

Conventional CVR mapping techniques require an explicit physiological maneuver, such as inhalation of CO2 (Yezhuvath et al., 2009; Spano et al., 2013; Liu et al., 2019), breath-holding (Geranmayeh et al., 2015), hyperventilation (Bright et al., 2009), or acetazolamide injection (Yonas et al., 1993; Ogasawara et al., 2002), which are cumbersome and may not be feasible in standard clinical settings, in large-scale studies, or in patients that cannot tolerate these physiological maneuvers. Unlike these conventional methods, RS-CVR mapping uses the spontaneous fluctuations in breathing pattern to measure CVR from typical resting-state BOLD data without needing additional challenges, making it a promising alternative CVR mapping approach to detect regional alterations in cerebrovascular function when conventional CVR mapping techniques are not feasible. By approximating the blood CO2 fluctuations using the filtered global BOLD signal, it also eliminates the need of EtCO2 recording as required by the conventional CVR mapping methods, allowing retrospective analysis of existing RS-fMRI data to obtain additional information of cerebrovascular function. In recent years, RS-CVR mapping has been applied in a number of clinical studies both prospectively and retrospectively, including in stroke (Taneja et al., 2019), Moyamoya disease (Liu et al., 2021), glaucoma (Chan et al., 2021), brain tumor (Yeh et al., 2022), and cognitive impairment (Ni et al., 2021, 2022), and has revealed good sensitivity in detecting disease-related CVR changes. Therefore, understanding the dependence of RS-CVR mapping on imaging parameters is a timely effort to facilitate broader application of this new technique.

Our results showed that the sensitivity of RS-CVR increases with larger acquisition voxel size, i.e., lower resolution. This is expected as lower resolution benefits from higher signal-to-noise ratio (SNR). However, even at the highest resolution we examined, at 2 × 2 × 2 mm^3^ without any smoothing in postprocessing, the resulting mean Z score across the brain was still higher than 4. After smoothing by a 4 mm FWHM kernel, the mean Z score of 2 × 2 × 2 mm^3^ was 7.14, which is more than three times of the typical cutoff Z score to define task activation area at individual subject level with the same acquisition resolution and 4 mm smoothness [e.g., Z > 1.96 (Barch et al., 2013)]. We also observed higher Z scores in gray matter than in white matter, consistent with all previous CVR studies. The lower sensitivity in white matter can be explained by longer arterial transit time and reactive time (Thomas et al., 2014), as well as lower SNR in white matter, compared to gray matter.

Unlike the dependence of sensitivity on imaging resolution, the accuracy of RS-CVR maps were found to be similar across different imaging resolutions, using CO2-CVR maps as the gold standard. This was still the case when applying different smoothness in postprocessing, even though larger smoothing kernel in postprocessing resulted in slight increase in overall spatial correlation between RS-CVR and CO2-CVR. With the smoothing kernel of 8 mm FWHM, the r values between RS-CVR and CO2-CVR of all three imaging resolutions were in the range of 0.67–0.91, which is considered strong correlation (Schober et al., 2018) and is consistent with those reported previously in healthy subjects and in patients with Moyamoya disease (Liu et al., 2017, 2021). This observation is important because most existing resting-state fMRI studies performed without simultaneous multi-slice acquisition used imaging resolutions around 2.5–3.5 mm. Since imaging resolution was found to have little effect on the accuracy of RS-CVR maps, this supports the retrospective analyses of existing resting-state fMRI data for CVR mapping.

Reproducibility of RS-CVR, assessed by comparing two halves of the resting-state scans, was also found to be independent of imaging resolution. The two RS-CVR maps of each imaging resolution demonstrated strong correlation, indicate good reproducibility at all three imaging resolutions. The r values of two RS-CVR maps were higher than those between RS-CVR and CO2-CVR. The r value is expected to increase when applying further smoothing in postprocessing. The spatial ICC of the RS-CVR maps (with no smoothing) were smaller than that of CO2-CVR map reported previously (0.93 ± 0.04) by Ravi et al. (2016) using 3.2 × 3.2 × 3.5 mm^3^ imaging resolution with 6 mm smoothing in postprocessing. Nonetheless, following the standard groupings of ICC as poor (<0.4), fair (0.41–0.59), good (0.60–0.74) or excellent (>0.75) reliability (Cicchetti, 2001), the spatial ICC of RS-CVR maps (0.78 or higher for all resolutions) can still be considered as excellent reproducibility.

Duration of the resting-state BOLD scan was found to have significant effects on the sensitivity but not on the accuracy of RS-CVR maps. This finding suggests that RS-CVR mapping may be applied to any resting-state BOLD data with a duration longer than 4 min without sacrificing the accuracy. But to ensure a relatively good sensitivity, the scan duration is expected to be 7 min or longer. This information can be used to guide the selection of existing resting-state BOLD data for retrospective analysis and also the planning of prospective resting-state BOLD scans. Although we have only examined resting-state BOLD data with a duration up to 10 min, we expect scans longer than 10 min will yield slightly higher sensitivity but similar accuracy of RS-CVR maps.

There are a few limitations of this study. First, the RS-CVR maps we obtained were in relative units, rather than in absolute units of percentage per millimeter of mercury CO2. Thus, this method is more suited for diseases in which CVR deficits are regional. Although recording of end-tidal CO2 during RS BOLD imaging will allow the absolute quantification of CVR, it will add to the complexity of the procedure. Second, we did not consider the voxel-wise CO2 bolus transit and response time which can also be a sensitive index of cerebrovascular diseases, especially in large vessel diseases. Previous work by our group and others have suggested good sensitivity of RS-CVR in detecting abnormal CVR regions without considering voxel-wise transit/delay (Taneja et al., 2019; Chan et al., 2021; Liu et al., 2021; Ni et al., 2021, 2022). There are a few advanced analysis methods to extract transit/delay maps from resting-state BOLD data, but it is beyond the scope of the present work. Third, the sample size of this study (*N* = 8) is small and only young healthy subjects (age 26.5 ± 5 years) were included. Although the CVR maps were compared within individual subjects, it is possible that the performance of RS-CVR may be different in older subjects or clinical cohorts. In our previous study (Liu et al., 2021), we have found slightly lower success rate of RS-CVR mapping in participants with Moyamoya diseases than that in healthy controls. Therefore, larger-scale studies with broader age ranges and in clinical populations could be performed in future to evaluate the spatial resolution-dependence of RS-CVR in an age-specific manner and in clinical settings.

## Conclusion

CVR maps obtained with resting-state BOLD fMRI revealed resolution-dependent sensitivity. However, even at a high resolution of 2 mm isotropic voxel size, the voxel-wise sensitivity is still greater than that of typical task-evoked fMRI. RS-CVR maps showed a high spatial correspondence with those obtained with standard CO2-inhalation based methods. These findings suggest that resting-state CVR can be applied at a similar resolution as state-of-the-art fMRI studies, which will broaden the use of CVR mapping in basic science and clinical applications including retrospective analysis of previously collected fMRI data.

## Data availability statement

The raw data supporting the conclusions of this article will be made available by the authors, without undue reservation.

## Ethics statement

The studies involving human participants were reviewed and approved by Institutional Review Board of the Johns Hopkins University School of Medicine. The patients/participants provided their written informed consent to participate in this study.

## Author contributions

PL and DJ contributed to the conception and design of the study. PL, LK, PJ, CX, and DJ participated in data collection. BH and LK performed data analysis, organized the database, and performed the statistical analysis. PL wrote the first draft of the manuscript. All authors contributed to manuscript revision, read, and approved the submitted version.
