# Supplementary material for: Dependence of resting-state-based cerebrovascular reactivity (CVR) mapping on spatial resolution
Source: Front Neuroimaging. 2023 Jun 26;2:1205459. doi: 10.3389/fnimg.2023.1205459 (PMC10406303; doi:10.3389/fnimg.2023.1205459)
Supplement: Supplementary file 1 [file Data_Sheet_1.docx]

Supplementary Material

Dependence of resting-state-based cerebrovascular reactivity (CVR) mapping on spatial resolution

Peiying Liu*, Beini Hu, Lincoln Kartchner, Parimal Joshi, Cuimei Xu, and Dengrong Jiang

*** Correspondence:** Peiying Liu: peiyingliu@som.umaryland.edu

## Supplementary Figures


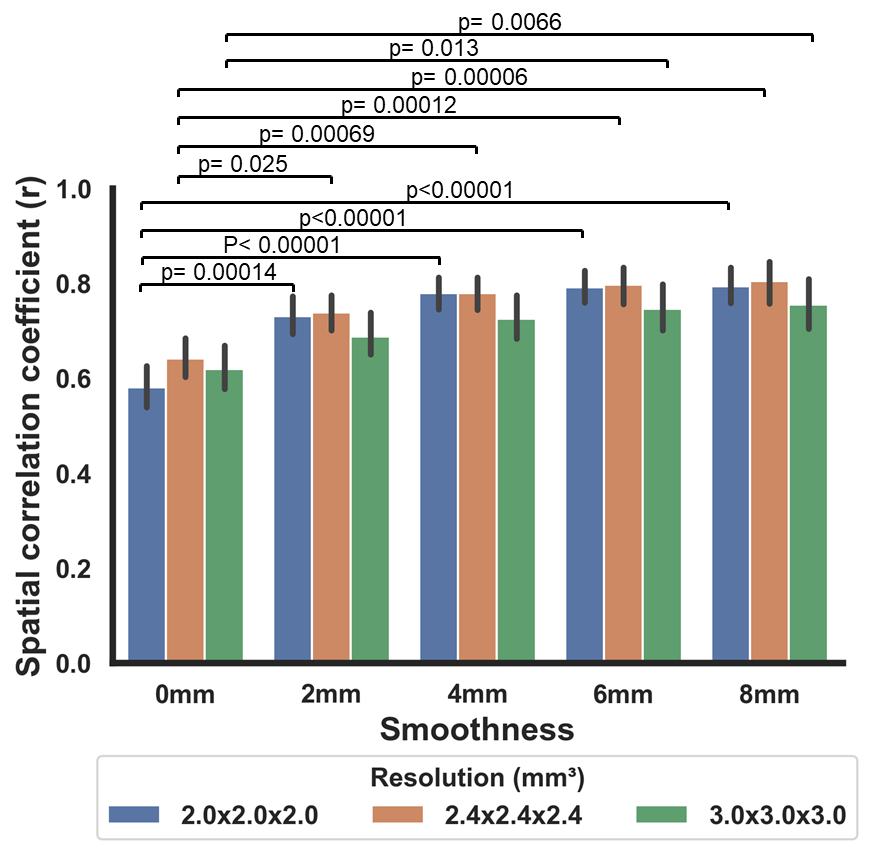


**Supplementary Figure 1.** Comparisons of spatial correlation coefficients between the RS-CVR maps and CO2 CVR maps at different smoothness in postprocessing.
